# Laboratory-based case finding using phosphate and alkaline phosphatase to identify genetic and nutritional rickets in children

**DOI:** 10.3389/fendo.2026.1899612

**Published:** 2026-09-28

**Authors:** Beatriz Domínguez-Sevillano, Mary Alejandra Ferrín-Ferrín, David Cembrero-Fuciños, José Carlos Moyano-Maza, M. Carla Criado-Muriel, Elena Marcos-Vadillo, Belén García-Berrocal, Pedro Gómez de Quero-Masía, Pablo Prieto-Matos, María Isidoro-García

**Affiliations:** 1 Servicio de Pediatría, Hospital Universitario de Salamanca, Salamanca, Spain; 2 Instituto de Investigación Biomédica de Salamanca, Salamanca, Spain; 3 Servicio de Análisis Clínicos, Hospital Universitario de Salamanca, Salamanca, Spain; 4 Unidad de Diagnóstico Avanzado de Enfermedades Raras de Castilla y León (DiERCyL), Hospital Universitario de Salamanca, Salamanca, Spain; 5 Departamento de Ciencias Biomédicas y del Diagnóstico, Universidad de Salamanca, Salamanca, Spain; 6 Departamento de Medicina, Universidad de Salamanca, Salamanca, Spain

**Keywords:** alkaline phosphatase, hypophosphatemia, hypophosphatemic rickets, *PHEX*, phosphate, nutritional rickets

## Abstract

**Introduction:**

Hypophosphatemic rickets is a rare disease characterized by renal phosphate loss, leading to chronic hypophosphatemia, elevated alkaline phosphatase, and skeletal symptoms that may remain unnoticed until advanced stages. Early diagnosis is essential because effective treatment is available to reduce complications.

**Objectives:**

To evaluate a laboratory-based case-finding strategy for identifying oligosymptomatic patients with hypophosphatemic rickets using phosphorus and alkaline phosphatase results in a pediatric population.

**Methods:**

A descriptive observational study was conducted in patients under 18 years of age at a tertiary care hospital between June 2021 and July 2025. Of 47,127 phosphorus or alkaline phosphatase measurements requested, 45,923 measurements corresponding to 28,045 tests from 12,513 patients were analyzed after exclusions. Test showing hypophosphatemia and/or elevated alkaline phosphatase were identified, and X-rays and clinical data were reviewed for classification.

**Results:**

A total of 22,860 phosphorus measurements and 23,063 alkaline phosphatase measurements were analyzed, with both parameters available in 17,878 laboratory tests. Hypophosphatemia was observed in 2,281 phosphorus measurements (9.98%), and elevated alkaline phosphatase in 3,301 measurements (14.3%). Fifty-five laboratory tests (0.31%) from 37 patients simultaneously met the criteria for hypophosphatemia (<−2 SD) and elevated alkaline phosphatase (>+2 SD); 56.8% were male, with a mean age of 6.5 ± 6.0 years. Following clinical review, 15 patients (40.5%) were classified as having a bone mineral metabolism disorder, including 10 with osteopenia of prematurity, three with nutritional rickets, one with a pathogenic variant in *PHEX*, and one with osteoporosis in the context of ReNU syndrome; 10 patients (27.0%) were classified in the acute/critical illness group and 12 (32.4%) in the other category. Radiographs were available in 30 patients, with findings consistent with rickets in three (10%). One previously unrecognized case of mild nutritional rickets was identified.

**Conclusions:**

Laboratory-based case finding using phosphorus and alkaline phosphatase may help identify pediatric bone metabolism disorders, including forms of rickets. Combined hypophosphatemia and elevated alkaline phosphatase may be useful for guiding etiological investigation. This approach may complement clinical evaluation and facilitate earlier diagnosis. Early identification is clinically relevant because treatments can modify disease course and reduce long-term complications in children with confirmed forms of rickets, when appropriate.

## Introduction

Rickets is a disorder of mineralization of the growth plate in the developing skeleton (1–4). Most cases of infantile rickets are caused by nutritional deficiencies, specifically vitamin D or calcium deficiency (5, 6). This type of rickets has been extensively studied, and there are international guidelines that clearly define its prevention, diagnosis, and treatment (6). In contrast, hereditary forms, such as X-linked hypophosphatemic rickets (XLH), continue to pose a diagnostic challenge, especially mild or atypical forms (7).

XLH is a genetic disorder caused by inactivating variants in the *PHEX* gene (OMIM*300550), which are associated with elevated levels of fibroblast growth factor 23 (FGF23). This excess of FGF23 leads to decreased tubular reabsorption of phosphate and inhibition of 1,25-dihydroxyvitamin D synthesis, resulting in chronic hypophosphatemia with defects in bone mineralization (1, 4, 7–12). The incidence is estimated at 3.9 per 100,000 live births, and the prevalence ranges from 1.7 to 4.8 per 100,000 inhabitants (1, 11).

Clinically, XLH manifests in early childhood with lower limb deformities, abnormal gait, disproportionate short stature, bone pain, and dental abscesses. In some cases, the presentation may be mild or subclinical, which can delay diagnosis even into adulthood, especially in women with mild forms (1, 7, 9–11, 13).

Typical biochemical findings include persistent hypophosphatemia, elevated alkaline phosphatase, low TmP/GFR, and inappropriately normal levels of 1,25-dihydroxyvitamin D, with parathyroid hormone (PTH) levels generally normal (7–10, 12, 14). In children, the combination of hypophosphatemia with elevated alkaline phosphatase constitutes a biochemical profile characteristic of rickets and has been proposed as a useful case-finding approach (2, 7).

However, in routine clinical practice, these findings often go unnoticed. A recent study based on laboratory databases showed that less than 5% of patients with hypophosphatemia underwent adequate etiological evaluation, and that through systematic review of laboratory data, it was possible to identify previously undiagnosed cases of XLH and other types of rickets (15).

Early diagnosis is clinically important, given that biochemical signs may precede clinical ones, especially in families with a history of XLH, and early initiation of treatment is associated with better growth outcomes and less severe deformities (16). Although standard treatment with oral phosphate and calcitriol had limited efficacy and side effects (primarily gastrointestinal), targeted therapies such as burosumab, a monoclonal anti-FGF23 antibody, have been shown to significantly improve biochemical parameters, radiological findings, and quality of life (1, 4, 7, 9, 10, 12, 14, 17).

Building on previous laboratory-based case-finding studies, we performed a proof-of-concept evaluation of a pediatric strategy combining age- and sex-adjusted phosphorus and alkaline phosphatase values with clinical review of patients showing a suggestive biochemical profile. The objective was to assess its potential utility for identifying rickets and other relevant disorders of bone and mineral metabolism.

## Materials and methods

A retrospective descriptive observational study was conducted in the pediatric population of a high complexity hospital during the period from June 2021 to July 2025. All laboratory tests performed in patients under 18 years of age during the study period that included serum phosphorus and/or alkaline phosphatase were included. A laboratory test was defined as a single blood sampling episode, which could contribute one or two measurements. Tests with incorrect patient identification or samples of insufficient quality or quantity were excluded. Repeated tests from the same patient were included in test-level analyses, whereas each patient was considered only once for clinical evaluation and diagnostic classification.

Phosphorus and alkaline phosphatase concentrations were determined using standard spectrophotometric techniques on an automated platform (Atellica^®^ Solution Analyzer CH-930, Siemens Healthineers Diagnostics). Inorganic phosphorus was measured using the phosphomolybdate method, and alkaline phosphatase using the P-nitrophenyl phosphate method with IFCC AMP buffer. Both assays were calibrated and controlled according to the manufacturer’s specifications and clinical laboratory standards.

To allow comparison across ages and sexes, phosphorus and alkaline phosphatase values were transformed into standard deviations (SD) using the laboratory’s internal reference tables, adjusted for age and sex. Hypophosphatemia was defined as a value below -2 SD and elevated alkaline phosphatase as above +2 SD, and the simultaneous presence of both abnormalities in a single determination was considered a “biochemical profile suggestive of rickets”. These predefined thresholds were selected to identify clearly abnormal age- and sex-adjusted biochemical profiles rather than to establish optimized diagnostic cutoffs. Height SD scores were calculated using the 2010 Spanish Growth Study reference standards, with short stature defined as height below −2 SD.

After identifying patients with a suggestive profile, their medical records were reviewed (clinical context and reason for workup, anthropometry, radiological and additional laboratory studies, and, when available, genetic study results) to help determine the cause of the abnormalities. When information was insufficient, the family was contacted to obtain relevant clinical data. Finally, the patients were classified into three clinical groups: (1) bone mineral metabolism disorder; (2) acute/critical context (e.g., admission/care for a severe acute condition potentially associated with transient phosphorus disturbances); and (3) other, for the remaining situations not fitting into the previous groups.

Osteopenia of prematurity was classified as such when documented in the medical record in preterm infants and supported by the available biochemical profile (phosphorus, alkaline phosphatase, calcium, and PTH). Nutritional rickets was diagnosed on the basis of compatible clinical and biochemical findings, including 25-hydroxyvitamin D, calcium, and PTH. The data were initially exported to Microsoft Excel and subsequently analyzed using IBM SPSS Statistics version 27. Quantitative variables were described as mean ± standard deviation or median and interquartile range, depending on the distribution (assessed using the Kolmogorov-Smirnov test); qualitative variables were expressed as absolute and relative frequencies. The comparison of proportions between groups was performed using chi-square (or Fisher’s exact test when appropriate), with p < 0.05 considered significant.

The study complied with the principles of the Declaration of Helsinki and Spanish data protection legislation. Clinical information was managed pseudonymously using a unique numerical identifier. The protocol was approved by the corresponding Health Area’s Committee for Medicines Research Ethics (protocol code PI 2025 04 1903).

## Results

During the study period (June 2021–July 2025), 47,127 phosphorus and/or alkaline phosphatase test results were recorded for individuals under the age of 18. After excluding misclassified tests or those of insufficient quality or quantity, 45,923 measurements were included, corresponding to 28,045 tests performed on 12,513 patients. Phosphorus was measured in 22,860 tests, alkaline phosphatase in 23,063, with both measurements present in 17,878 tests from 9,736 patients.

The mean age at the time of sample collection was 11.46 ± 4.83 years; 51.4% of the tests were performed on girls.

In the tests measuring phosphorus, the mean concentration was 4.77 ± 0.92 mg/dL, corresponding to -0.45 ± 1.85 SD relative to the laboratory reference values. Hypophosphatemia was observed in 2,281 tests (9.98%).

In tests measuring alkaline phosphatase, the mean concentration was 214.5 ± 149.9 U/L, corresponding to 0.32 ± 2.91 SD. Elevated alkaline phosphatase was observed in 3,301 tests (14.3%).

The proportion of samples with low phosphorus was 10.2% in the emergency department, 7.5% in outpatient clinics, 7.2% in primary care, 3.5% in inpatient care, and 6.3% in other services (p < 0.001). The proportion of samples with elevated alkaline phosphatase was 25.9% in inpatient care, 5.8% in the emergency department, 4.9% in outpatient clinics, 1.8% in primary care, and 12.7% in other services (p < 0.001).

Of these 17,878 test results with simultaneous measurements, 55 (0.31%) met the criteria for both hypophosphatemia and elevated alkaline phosphatase. These 55 test results corresponded to 37 patients. The characteristics of this subgroup are presented in **Supplementary Material 1**.

The 55 test results showing hypophosphatemia and elevated alkaline phosphatase corresponded to 37 patients. Clustering of results was observed in some cases: one patient had 8 test results with both abnormalities, another had 4, three patients had 3 test results, and two patients had 2 test results.

Of the 37 patients, 21 (56.8%) were male. The mean age was 6.5 ± 6.0 years (median 5.5 [0.21–11.49] years). Twelve patients (32.4%) were 6 months of age or younger, all of whom had a history of prematurity. The laboratory values for these patients were phosphorus 3.5 [2.9 to 4] mg/dL and -2.4 [-3.1 to -2.1] SD and alkaline phosphatase of 424.3 [355.1 to 579.2] U/L and 3.7 [2.5 to 7.4] SD (**Table 1**).

**Table 1:** Comparison of laboratory tests with simultaneous determination of phosphorus and alkaline phosphatase according to biochemical profile (P < −2 SD and ALP > +2 SD).

Variable	Non-suggestive profile N = 17,823	ALP > +2 SD and P <- 2 SD N = 55	p
Age (years)	12.7 [8.4 to 15.5]	8.7 [2.2 to 16.8]	< 0.001
Sex (female/male)	51.4/48.6%	43.2/56.8%	> 0.05
P (mg/dL)	4.76 [4.28 to 5.23]	3.5 [2.9 to 4]	< 0.001
P (SD)	-0.8 [-1.4 to -0.3]	-2.4 [-3.1 to -2.1]	< 0.001
ALP (U/L)	208.4 [131.7 to 272.7]	424.3 [355.1 to 579.2]	< 0.001
ALP (SD)	-0.32 [-1.1 to 0.6]	3.7 [2.5 to 7.4]	< 0.001

Data are expressed as median [IQR]. Comparisons were performed using the Mann–Whitney test; proportions were analyzed using the χ²/Fisher test. For the suggestive biochemical profile group, sex distribution is reported at the patient level (n = 37), with each patient counted once; all other variables are reported at the test level.

This subgroup of 37 patients constituted the series on which the detailed clinical evaluation was performed. In five patients, diagnostic uncertainty persisted after the initial evaluation, so the study was expanded to include additional laboratory tests and/or genetic testing. The expansion was completed in 4 of 5 cases. In the other case, which presented with an isolated biochemical abnormality (Patient 26; **Supplementary Material 1**), it was not possible to complete the expansion because subsequent contact with the family could not be established.

Finally, radiographs were obtained in 30 of 37 patients, with abnormalities consistent with rickets observed in 3 of 30 (10%, **Figure 1**). Height data were available for 34 patients, with 8 having a height below -2 SD. Additional laboratory tests were performed in 31 patients, and genetic testing (massive sequencing or other genetic tests) was conducted in 8 (**Table 2**).

**Figure 1:**
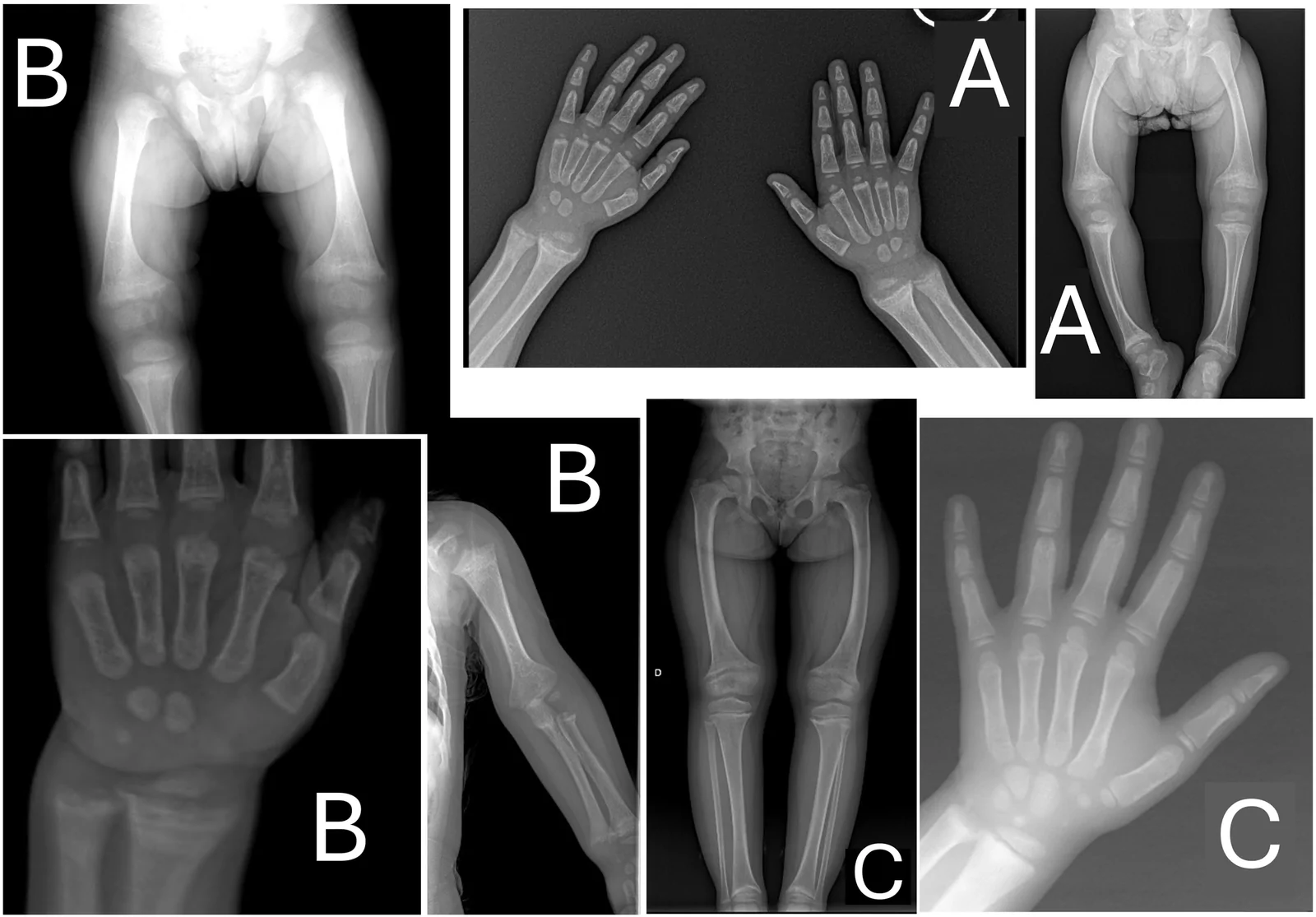
Radiographs of the three patients with metaphyseal abnormalities consistent with rickets. **(A, B)** Nutritional rickets. **(C)** X-linked hypophosphatemic rickets (XLH).

**Table 2:** Genetic findings in the 8 patients who underwent genetic testing.

Patient number	1	2	5	8	16	17	18	28
Genetic test	Sanger Exome	Exome	Exome	Exome	Exome Genome CGH-array	Exome	Sanger	Exome
Gene	*RNU4-2* *TRPV6*	*VDR*	*PHEX*	*-*	*-*	*NF1*	*ACTG2*	–
Variant	NR_003137.3 n.76C>T and NM_018646 c.2057G>A p.Arg686His and NM_018646 c.1936C>T p.Arg646Trp	NM_001364085.2 c.2T>C p.Met1Thr Homozygous	NM_000444.5 c.1530dupC p.Lys511Glnfs*7	No findings	GRCh37/hg19 Chromosome 17 Deletion 46212366-47808056 Del 17q21.32-q21.33	NM_001042492.3 c.6772C>T p.Arg2258Ter	NM_001615.3 c.770G>A p.Arg257His	No findings
Pathogenicity Criteria (ACMG)	PS4, PM2, BP7, PP5 Likely pathogenic; PM2, BP4 Uncertain significance; PM2 Uncertain significance	PVS1, BA1, BS2, BP6 Benign	PP4, PVS1, PM2 Pathogenic	–	–	PS4, PVS1, PM2, PP5, PP4 Pathogenic	PS4, PP4, PM2, PM5, PP3, PM6, PP2, PP5 Pathogenic	–
Diagnosis	ReNU syndrome	Predisposition to nutritional rickets	Hypophosphatemic rickets	–	Contiguous gene syndromes on chromosome 17	Neurofibromatosis type 1	Berdon syndrome	–

Of the patients with suggestive biochemical profiles, following clinical evaluation they were classified into the following groups: abnormal bone mineral metabolism (n = 15), acute/critical condition (n = 10), and others (n = 12).

Of the 15 patients (40.5%) classified in the mineral/bone disorder category, 10 had osteopenia of prematurity, three had deficiency rickets, one had XLH rickets, and one had osteoporosis in the context of a ReNU syndrome. The clinical, biochemical, radiological, and genetic characteristics of these four patients diagnosed with rickets are summarized in **Table 3**.

**Table 3:** Data on children with deficiency rickets or XLH (*PHEX*).

Patient number	Patient 2	Patient 3	Patient 4	Patient 5
Diagnosis	Severe nutritional rickets	Mild nutritional rickets	Severe nutritional rickets	X-linked hypophosphatemic rickets
Ca (mg/dL)	9	9	9.3	9.7
P (mg/dL)	2.9	4	3.3	2.4
Mg (mg/dL)	2.3	2.2	2.19	1.8
Cr (mg/dL)	0.14	0.36	0.14	0.32
PTH (pg/mL)	206	105	282	44.6
ALP (U/L)	939	611	1,700	521
25-hydroxyvitamin D (ng/mL)	15.8	8.7	5	23.2
1,25-dihydroxyvitamin D (pg/mL)	181	–	110	62
Ionized calcium (mmol/L)	1.1	1.07	1.1	1.09
Urinary creatinine (mg/dL)	27	–	68	25
Urinary phosphorus (mg/dL)	23.1	–	65.4	55.8
Urinary calcium (mg/dL)	<0.8	–	1	4.3
Tubular reabsorption of phosphate (%)	95.9	–	95.9	70.2
FGF23 (RU/mL)	–	–	1,163	–
Genes	*VDR:* NM_001364085.2: c.2T>C (homozygous)	–	–	*PHEX:* NM_000444.5: c.1530dupC (heterozygous)

The patient diagnosed with XLH rickets had a pathogenic variant in *PHEX* (NM_000444.5: c.1530dupC) and type 1 diabetes mellitus. Although the diagnosis of XLH was already established, a pre-diagnostic blood test met the predefined biochemical criteria and was identified by the case-finding strategy.

Among the three cases classified as deficiency rickets, two presented with severe clinical symptoms. In one of these cases, given elevated levels of 1,25-dihydroxyvitamin D with 25-hydroxyvitamin D not clearly decreased, an exome study was performed to rule out vitamin D resistance. The study identified the variant NM_001364085.2: c.2T>C (p.Met1Thr) in homozygosity in *VDR*, classified as benign (FokI polymorphism).

Both XLH rickets and severe deficiency rickets had already been diagnosed, while mild rickets was a new diagnosis. All previously diagnosed cases of rickets met the predefined biochemical criteria. In addition, the strategy identified one previously unrecognized case of mild nutritional rickets.

Finally, one patient was identified with impaired bone metabolism and a syndromic phenotype featuring intellectual disability. Genetic testing revealed two variants in *trans* in *TRPV6* (NM_018646: c.2057G>A and c.1936C>T), and a targeted Sanger sequencing study demonstrated the *de novo* pathogenic variant *RNU4-2* (NR_003137.3): n.76C>T. The combination of these findings was considered consistent with the patient’s clinical presentation.

Ten patients (27.0%) were classified in the acute systemic disease/critical condition category, including cases of sepsis or extreme severity, severe liver disease, and intestinal failure. In all these cases, the biochemical abnormalities were transient and normalized in subsequent tests, with no clinical or radiological findings consistent with structural bone disease.

The remaining 12 (32.4%) were classified as physiological/other, a category that included adolescent pregnancy and transient laboratory abnormalities without evidence of structural bone disease. Patients with non-primarily bone-related comorbidities predominated, including three cases of type 1 diabetes mellitus (a condition also present in the patient with XLH rickets), as well as isolated cases of hypothyroidism, intellectual disability, or obesity. In this group, abnormalities in phosphorus and alkaline phosphatase levels corresponded to isolated findings or transient conditions (such as pregnancy) without clinical or radiological data suggesting bone disease.

## Discussion

The main objective of this study was to assess whether a laboratory-based case-finding strategy using phosphorus and alkaline phosphatase in pediatric laboratory tests at a tertiary care hospital can identify cases of genetic and deficiency-related rickets, as well as other relevant bone and mineral disorders. Using the established criteria (phosphorus < -2 SD and alkaline phosphatase > +2 SD), the known case of XLH and the previously diagnosed cases of severe deficiency rickets were detected. In addition, osteopenia in preterm infants, one case of mild deficiency rickets, and one patient with bone disease and genetic findings in *TRPV6* and *RNU4–2* were identified.

These findings should be interpreted as a proof-of-concept. Although only one previously unrecognized case of rickets was identified, the strategy also detected pre-diagnostic laboratory tests from the patient with XLH that already fulfilled the predefined biochemical criteria, suggesting that it could have prompted an earlier diagnostic evaluation. Given its simplicity and reliance on routinely available laboratory data, validation in additional centers is warranted to assess its reproducibility and incremental diagnostic yield.

Taken together, these data suggest that laboratory-based case finding applied to existing laboratory tests may be a useful tool for identifying patients who are candidates for specific evaluation. However, its performance is limited; it depends on the tests requested in routine clinical practice, uses extreme cutoff points (with possible under detection of mild forms), and detects biochemical abnormalities in contexts such as prematurity or systemic disease, where the profile does not always reflect primary rickets.

The literature agrees that XLH rickets is underdiagnosed and that diagnostic delays are common, even in healthcare systems with easy access to laboratory tests and imaging studies (1–3, 14). In our series, the fact that the known case of XLH rickets was identified by the case-finding strategy supports the notion that the combination of hypophosphatemia and elevated alkaline phosphatase is a good starting point for identifying patients who should undergo targeted clinical and radiological evaluation. Furthermore, the identified case was a girl, whereas in boys the phenotype and biochemical abnormalities are typically more evident (7, 8, 14), which could reduce the likelihood of unidentified affected boys in our case-finding analysis. Nevertheless, only one pediatric case of XLH was identified in our cohort. Given the selected study population and retrospective design, this observation cannot be directly compared with published population-based prevalence estimates (3, 8, 18). Our study was limited to individuals under 18 years of age and to laboratory tests requested in routine clinical practice, precluding any formal estimate of incidence or prevalence.

In our cohort, three cases of deficiency rickets were also identified, consistent with the literature describing a resurgence of this condition in developed countries, particularly in children with risk factors such as restrictive diets, limited sun exposure, or exclusive breastfeeding without vitamin D supplementation (6), and in the period following lockdowns associated with the COVID-19 pandemic (19, 20). In one of these patients, a variant was also identified that did not in itself explain the clinical presentation (FokI polymorphism in *VDR*), although it has been linked to susceptibility to deficiency rickets by altering the start codon and generating a protein three amino acids shorter (21, 22). These findings reinforce the need to maintain a high clinical suspicion, even in contexts where adequate nutritional status is initially presumed.

A relevant finding was the case of a child with bone involvement and a biochemical profile consistent with rickets, in whom two variants of uncertain significance were identified in *TRPV6*, a gene described as a rare cause of rickets due to impaired intestinal calcium absorption (23), as well as ReNU syndrome caused by a pathogenic variant in *RNU4-2* (24). Although the phenotype bears some resemblance to what has been reported for *TRPV6*, the fact that these are variants of uncertain significance prevents us from conclusively attributing the bone abnormality to this mechanism. Furthermore, the patient presents with osteoporosis in addition to ReNU syndrome with severe disability and very limited mobility; therefore, it is possible that a significant portion of the bone involvement is secondary to immobility and the underlying neurological condition, rather than to primary rickets caused by *TRPV6*.

Our results show that the profile of hypophosphatemia with elevated alkaline phosphatase is not specific to XLH rickets, but also occurs in cases of secondary bone disease and severe systemic illness. A significant proportion of children in the bone disease group had osteopenia of prematurity, in the context of extreme prematurity and severe neonatal pathology, reflecting this group’s vulnerability to mineral depletion and necessitating the interpretation of biochemical findings with consideration of gestational age and nutritional history. Similarly, marked abnormalities in phosphorus and alkaline phosphatase were observed in patients with severe systemic disease (severe liver disease, sepsis, and intestinal failure), in whom the laboratory profile likely reflects the baseline condition. Consistent with reports from other series, elevated alkaline phosphatase in childhood is a relatively common and nonspecific finding, related in part to bone growth itself, and its diagnostic value increases when associated with marked hypophosphatemia and confirmed by repeated measurements (5).

An additional observation in our cohort was the repeated occurrence of type 1 diabetes mellitus in the group of patients with low phosphorus levels, including the case of XLH rickets. In several children with diabetes, episodes of hypophosphatemia were observed without clinical or radiological findings consistent with rickets, possibly related to metabolic control, osmotic diuresis, or intercurrent conditions that we were unable to demonstrate. Similarly, we identified abnormalities in phosphorus and alkaline phosphatase levels during adolescent pregnancy. Pregnancy is associated with physiological adaptations in mineral metabolism and an increase in total alkaline phosphatase, largely attributable to the placental isoenzyme rather than to primary bone disease (25). In both scenarios, the biochemical profile alone lacks specificity for rickets and underscores the need to integrate the interpretation of phosphorus and alkaline phosphatase with the clinical history, nutritional status, growth, and, when appropriate, radiological assessment.

From a methodological standpoint, the thresholds were defined to capture clearly altered profiles, rather than to establish optimized cutoff points. Because diagnostic verification was not systematically performed in patients who did not meet the biochemical criteria, this study was not designed to estimate diagnostic sensitivity, specificity, or predictive values. Nevertheless, all previously diagnosed cases of rickets represented in the study population were retrieved by the case-finding strategy, and one additional mild case was identified. The simplicity of the approach makes it readily applicable to routine laboratory data. An automated laboratory alert triggered by the simultaneous presence of phosphorus below the age and sex specific reference range and alkaline phosphatase above the corresponding upper reference limit could prompt targeted clinical review.

This study has several limitations. First, it is a single-center retrospective analysis influenced by routine clinical practice: only children for whom phosphorus and/or alkaline phosphatase levels were measured were included, so it is likely that some patients with rickets were not identified by our approach. Although the diagnostic work-up was not standardized across patients, the medical records of all patients meeting the biochemical criteria were systematically reviewed to assess the basis of the recorded diagnosis. When the available information was considered insufficient, additional biochemical and/or genetic investigations were initiated; however, these could not be completed in all cases because of the retrospective design and inability to recontact some families. Second, the case-finding strategy was based on predefined extreme cutoff points (phosphorus < -2 SD and alkaline phosphatase > +2 SD), without formal statistical optimization, which may fail to identify milder forms of rickets that do not reach these thresholds. External validity may be limited by differences between centers in laboratory reference ranges, test-ordering practices, patient characteristics, and access to diagnostic evaluation. Validation in independent pediatric cohorts will therefore be needed to assess generalizability.

Overall, our results show that a simple laboratory-based case-finding approach, based on the combination of marked hypophosphatemia and elevated alkaline phosphatase in routine pediatric laboratory tests, allows for the identification of the previously diagnosed XLH case and the detection of other clinically relevant forms of rickets and bone diseases. However, this approach detects a considerable number of patients with abnormal test results in the context of extreme prematurity, severe systemic disease, pregnancy, or type 1 diabetes mellitus, in whom the biochemical profile may primarily reflect the baseline condition rather than primary rickets. The challenge is not only to extend this strategy to other hospitals but also to refine it by systematically incorporating clinical, growth, and radiological information, so that laboratory-based case finding serves as an initial tool to identify patients requiring further clinical evaluation rather than as a diagnostic procedure in itself. Given that a substantial proportion of the abnormal profiles were concentrated among preterm infants and referrals from Neonatology, a possible optimization of the case-finding strategy would be to analyze this group separately using criteria focused on preterm osteopenia, reserving the main algorithm for the non-neonatal population.

In conclusion, our laboratory-based case-finding strategy identified the known case of XLH and other relevant forms of rickets and bone diseases, while also detecting biochemical abnormalities associated with prematurity, severe illness, pregnancy, or diabetes. This simple approach may help identify patients requiring further clinical evaluation, although its utility should be confirmed in prospective studies.

## Data Availability

The datasets presented in this article are not readily available because they contain sensitive clinical and laboratory data from pediatric patients, and their public disclosure could compromise participant confidentiality. Requests to access de-identified data should be directed to Pablo Prieto-Matos (pprieto@saludcastillayleon.es) and will be considered in accordance with applicable ethical and data-protection requirements.
